# En Bloc Multivisceral Resection and Portal Vein Thrombectomy for Metastatic Solid Pseudopapillary Neoplasm of the Pancreas: A Case Report

**DOI:** 10.7759/cureus.111380

**Published:** 2026-06-23

**Authors:** Jorge A Garcia Garza, Andrea N Garza Cisneros, Manuel A Medellin Vazquez, Bianca S Garcia Beattie, Benjamin Moya Leal, Jorge Andrés Cardoza Soria, Eduardo Flores-Villalba

**Affiliations:** 1 General Surgery, Hospital Regional de Monterrey Instituto de Seguridad y Servicios Sociales de los Trabajadores del Estado (ISSSTE), Monterrey, MEX; 2 General Medicine, Universidad de Monterrey, Monterrey, MEX

**Keywords:** en bloc surgery, liver metastasis tumor, portal vein thrombectomy, rare pancreatic tumor, solid pseudopapillary neoplasm of pancreas

## Abstract

Solid pseudopapillary neoplasms (SPNs) of the pancreas are rare tumors that typically exhibit a low malignant potential and an indolent clinical course; however, up to 10-15% of cases can manifest aggressive features, including vascular invasion and synchronous distant metastases. We report the case of a patient diagnosed with an aggressive pancreatic SPN associated with extensive local invasion, a 5 cm portal vein tumor thrombus, and synchronous hepatic metastases. The patient underwent an extensive, aggressive multi-visceral resection encompassing a distal pancreatectomy, splenectomy, superior hemigastrectomy, distal esophagectomy, and a portal vein thrombectomy with primary vascular repair. Follow-up imaging demonstrated stable hepatic disease with an increase in cystic-appearing lesions, while follow-up laboratory evaluations confirmed the complete normalization of biochemical and hematological parameters. Clinically, the patient experienced a satisfactory postoperative course, presenting with no compromise in nutritional status, adequate tolerance of oral intake, and optimal functional capacity, with an Eastern Cooperative Oncology Group (ECOG) performance status of 0, performing all activities of daily living autonomously and without limitations. This case underscores that aggressive multi-visceral surgical resection with cytoreductive intent is a technically feasible and highly beneficial strategy for metastatic SPN, as it can favorably modify the disease's biological behavior, reduce overall tumor burden, and achieve remarkable clinical and functional stability.

## Introduction

Pancreatic tumors encompass a heterogeneous spectrum of neoplasms with distinct biological profiles and prognoses, ranging from aggressive pancreatic adenocarcinomas to low-grade malignant neoplasms, such as solid pseudopapillary neoplasms (SPN) of the pancreas [1].

SPN of the pancreas, historically designated as Frantz's tumor, is an uncommon entity first characterized by Virginia Kneeland Frantz in 1959 [2]. It accounts for approximately 1% to 3% of all pancreatic malignancies and exhibits a marked predominance in young females [3,4]. Clinically, SPNs are characterized by an insidious growth pattern and, in many cases, an indolent biological course with low malignant potential. Nonetheless, up to 15% of cases can manifest aggressive features, including local invasion of adjacent structures or synchronous metastatic disease, predominantly targeting the liver, at the time of initial diagnosis [5,6].

Surgical resection with negative margins remains the cornerstone of management for SPNs, even in the setting of local invasion or metastatic dissemination, as multiple retrospective series have reported 5-year and 10-year disease-free survival rates exceeding 89-96% following aggressive surgical intervention [5,7]. Furthermore, in studies evaluating patients with resectable metastases, cytoreductive surgery has been shown to reduce tumor burden, alleviate mass effect symptoms, and potentially enhance overall survival, even in the presence of hepatic metastases [8].

We report the case of a 35-year-old female presenting with a large-volume SPN characterized by extensive vascular invasion and documented hepatic metastases. The patient underwent an aggressive en bloc multivisceral resection of the primary tumor combined with a portal vein thrombectomy with therapeutic intent, aimed at mitigating the malignant biological behavior of the disease and optimizing the global oncological prognosis.

## Case presentation

A 35-year-old female with no significant past medical history presented with a clinical onset nine months prior to surgical intervention. The initial presentation was characterized by melena and acute anemic syndrome; on physical examination upon admission, the patient exhibited marked mucocutaneous pallor and tachycardia, consistent with acute anemic syndrome, requiring emergency department management with blood product transfusions. Abdominal palpation revealed a firm, mildly tender mass located in the epigastrium and left hypochondrium, with no evidence of peritoneal irritation. Upon directed anamnesis, the patient noted early satiety and unintentional weight loss. Following upper endoscopy and colonoscopy, which yielded unremarkable findings, diagnostic imaging studies were performed during her hospitalization.

A thoraco-abdominal CT (Figure 1) revealed pulmonary parenchyma to be free of nodules or suspicious masses. In the liver, multiple irregularly shaped, poorly defined lesions were identified in a random distribution, demonstrating enhancement following intravenous contrast administration; the largest lesions were localized in segment VIII (9.5 x 8.9 x 8.7 cm) and segment V (10.3 x 8.3 cm). In the pancreatic body, an irregular mass extending into the tail was identified, measuring 9.3 x 12 x 9.7 cm, with involvement of the splenic vein and artery. Additionally, a luminal filling defect was documented within the portal vein, extending into the right portal branch, compatible with a tumor thrombus. 

**Figure 1:**
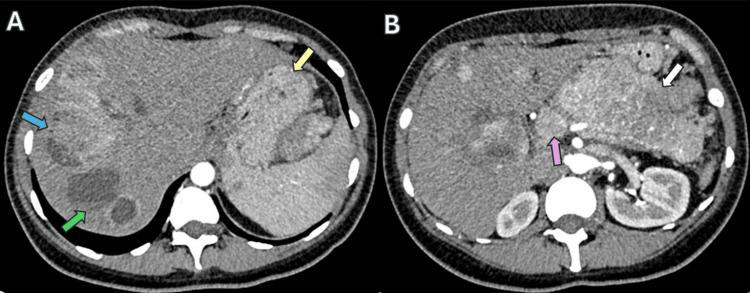
Initial CT scan (A) Preoperative axial CT image demonstrating poor contrast enhancement within the metastatic hepatic lesions. (B) Axial CT image showing the large, contrast-enhancing primary pancreatic mass causing compression and displacement of adjacent structures, alongside associated portal vein dilation. *Key: Light blue arrow: metastatic solid lesion; light green arrow: cystic liver lesion; white arrow: primary pancreatic tumor; yellow arrow: stomach; light purple arrow: portal vein.*

Histopathology

A percutaneous core needle biopsy (Trucut) of the liver reported a solid pseudopapillary neoplasm of the pancreas. The immunohistochemical profile confirmed the diagnosis, demonstrating positivity for E-cadherin and β-catenin, with a Ki-67 cell proliferation index of 1%.

Surgical approach

An exploratory laparotomy was performed, revealing hepatomegaly secondary to multiple solid, rounded lesions distributed across both hepatic lobes (the largest measuring 10 cm). An irregular, stony-textured tumor measuring 15 cm was identified in the pancreatic body and tail, displacing the stomach anterosuperiorly, and the tumor exhibited firm and friable adhesions to the posterior aspect of the gastric body, fundus, and cardia. Due to this extensive local involvement, a superior hemigastrectomy and distal esophagectomy were necessitated to achieve complete tumor clearance, followed by an esophagogastric anastomosis. The pancreatic neck, head, and uncinate process were free of macroscopic disease, allowing for preservation of the pancreatic head without compromising an R0 resection. Tumor thrombosis measuring 5 cm within the portal vein and firm splenic adhesions were confirmed.

Figure 2 shows the procedures performed.

**Figure 2:**
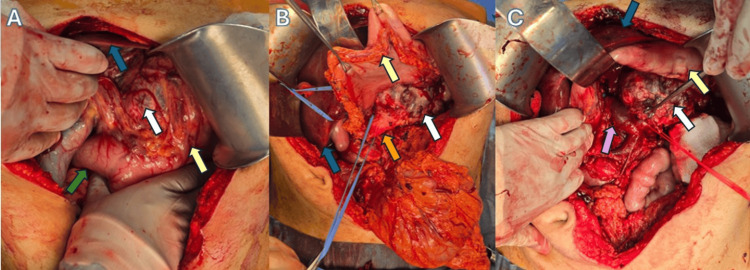
Intraoperative findings (A) Initial surgical approach demonstrating significant gastric displacement due to a large tumor mass adjacent to the lesser omentum. (B) Isolation of critical structures utilizing surgical loops (blue vessel loops around the hepatic hilum and pancreatic neck region), followed by omentectomy and a Kocher maneuver to completely expose the pancreatic tumor. (C) Exposure of the superior mesenteric vein using a red vascular loop, revealing significant dilation of the portal vein secondary to a 5 cm tumor thrombus. *Key: White arrow: pancreatic tumor; yellow arrow: stomach; light purple arrow: portal vein; blue arrow: liver; green arrow: duodenum; orange arrow: pancreatic neck.*

An omentectomy with resection of the hepatogastric and hepatoduodenal ligaments was performed. A distal pancreatectomy using a linear stapler and concomitant splenectomy was subsequently performed.

Due to this extensive local involvement, a superior hemigastrectomy and distal esophagectomy were performed using 80 mm linear staplers due to firm adhesions, achieving complete liberation of the specimen.

Portal vein thrombectomy following vascular clamp placement: a 3 cm incision was performed, followed by portal vein closure with 6-0 Prolene (running suture), achieving adequate hermeticity.

Gastroesophageal anastomosis with a 29 mm circular stapler, via a prior 4 cm incision on the anterior gastric wall and exteriorization through the posterior wall, along with anvil placement in the distal esophagus; primary closure of the anterior incision was completed using an 80 mm linear stapler.

Placement of two Blake-type drains: the right drain was positioned near the esophagogastric anastomosis, extending into the right parietocolic gutter, and the left drain was placed in the left parietocolic gutter.

Final histopathology

Macroscopic evaluation of the resected multivisceral specimen revealed a mass in the pancreatic body and tail measuring 8.5x7.5x6.5 cm. The tumor was surrounded by a whitish capsule and exhibited a heterogeneous, multinodular cut surface with light brown, dark brown, and reddish-brown areas, consistent with internal degeneration. The mass demonstrated direct adherence to the perisplenic soft tissues, the splenic hilum, and the posterior wall of the stomach along the greater curvature, achieving negative proximal and distal gastric resection margins (0.5 cm and 0.7 cm, respectively). Additionally, assessment of the portal vein tumor thrombus confirmed a 4.5x1.2x1.0 cm heterogeneous soft tissue mass. Microscopic examination on routine staining revealed a highly cellular neoplasm exhibiting a predominantly solid architectural pattern. Given the morphological overlap often seen in solid pancreatic neoplasms on standard evaluation, the definitive diagnosis of solid pseudopapillary neoplasm was firmly sustained by the immunohistochemical profile of being diffusely positive for E-cadherin and β-catenin, which remains the diagnostic gold standard for this clinical entity.

Postoperative evolution and follow-up

Using the pancreatic cancer TNM classification (American Joint Committee on Cancer [AJCC] 8th edition), the tumor was staged as T3N0M1. The patient experienced a favorable clinical course during a 10-day hospitalization, characterized by progressive clearing of the drains with output declining to <50 mL/day and adequate oral intake tolerance, and was subsequently discharged for outpatient follow-up by General Surgery and Medical Oncology services.

Regarding adjuvant treatment, the case was evaluated by a multidisciplinary tumor board. Given the characteristic chemoresistance of SPNs and the lack of standardized systemic therapies for metastatic presentations, a strategy of strict clinical and radiological surveillance was adopted rather than empirical systemic therapy.

Follow-up imaging

Chest and abdominal CT demonstrated pulmonary parenchyma with bilateral basal segment consolidations. The liver showed multiple diffuse hypodense lesions compatible with metastatic disease. The stomach exhibited postoperative changes within the gastric body along with the presence of anastomotic staples.

The patient experienced a satisfactory postoperative evolution, characterized by clinical and hemodynamic stability. She presented with no nutritional status compromise, demonstrating adequate oral intake tolerance and preserved intestinal transit. Follow-up laboratory evaluations confirmed the normalization of biochemical and hematological parameters. Functionally, the patient maintained an ECOG performance status of 0, performing activities of daily living autonomously and without limitations.

Recent control CT

The pulmonary parenchyma showed no areas of alveolar occupation or nodular lesions. The liver exhibited multiple irregular, poorly defined masses within segment V (7.6 x 6.7 cm) and segment VIII (5.4 x 3.8 cm), with an increase in areas of cystic appearance. The stomach demonstrated postoperative changes with a visible stapling line within the gastric body region (Figure 3).

**Figure 3:**
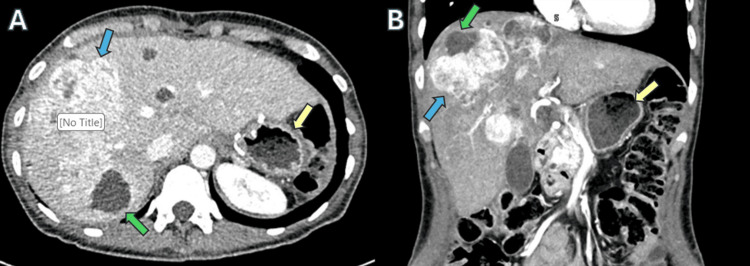
Follow-up CT scan (A) Axial view and (B) coronal view of the follow-up CT scan demonstrating stable metastatic disease, characterized by an increase in areas of cystic transformation within the hepatic lesions without a significant increase or decrease in overall tumor dimensions. Postoperative changes within the gastric body are also well visualized. *Key: Light blue arrow: metastatic solid lesion; light green arrow: cystic-appearing liver lesion; yellow arrow: stomach (postoperative status).*

Table 1 presents the comparative analysis of biochemical and hematological parameters during the evolutionary follow-up, encompassing the postoperative period and the clinical reevaluation at six months following the surgical intervention.

**Table 1:** Comparative analysis of hematological and biochemical parameters during clinical follow-up Laboratory evaluations were obtained postoperatively and at the six-month clinical follow-up. Initial abnormalities reflect the acute surgical stress response and baseline tumor mass effect, which subsequently show progressive improvement in most parameters with persistent mild hypoproteinemia and hyperglycemia.

Laboratories	Postoperative	6 Months Later	Units	Reference Range
White Blood Cells (WBC)	14.78	4.88	10^3/µL	4.50 – 11.00
Neutrophils	88.2	34.2	%	40.0 – 70.0
Lymphocytes	6.6	48.6	%	20.0 – 45.0
Hemoglobin	7.9	13	g/dL	12.0 – 15.5
Hematocrit	21.7	40	%	36.0 – 48.0
Platelets	184	422	10^3/µL	150 – 450
Glucose	135	144	mg/dL	70 – 100
Urea	37	13	mg/dL	15 – 45
Creatinine	1.1	0.4	mg/dL	0.6 – 1.1
Direct Bilirubin	0.1	0.1	mg/dL	0.0 – 0.3
Indirect Bilirubin	0.4	0.4	mg/dL	0.2 – 0.8
Total Bilirubin	0.5	0.5	mg/dL	0.3 – 1.2
Alanine Aminotransferase (ALT/SGPT)	58	57	U/L	< 33
Aspartate Aminotransferase (AST/SGOT)	182	53	U/L	< 32
Alkaline Phosphatase (ALP)	154	262	U/L	35 – 105
Albumin	2.4	3.1	g/dL	3.5 – 5.2

## Discussion

Solid pseudopapillary neoplasms (SPN) of the pancreas are traditionally characterized as tumors of low malignant potential with a favorable clinical outcome following complete surgical resection [9]. However, the current case illustrates that SPNs can present with highly aggressive features, such as extensive local invasion, vascular involvement, and metastatic disease, aligning with findings observed in a small minority of patients [5].

Role of surgery in SPN with malignant features

In contrast to most aggressive pancreatic malignancies, SPN demonstrates a highly favorable response to surgical intervention, even in the context of metastatic dissemination [8,10]. Evidence shows that complete R0 resection with negative margins is considered curative in the majority of cases without distant metastatic disease; furthermore, patients exhibiting malignant features, including local invasion or distant metastases, frequently achieve favorable long-term survival when undergoing aggressive surgical management with either curative or palliative intent [5,8].

In a retrospective series evaluating 32 patients with SPN who underwent surgical resection, only a small subset presented with hepatic metastases or adjacent organ invasion; despite these aggressive features, the 5- and 10-year disease-free survival rates were 96.5% and 89.6%, respectively, following R0 resection [5]. Similarly, a broader analysis demonstrated 5-year survival rates of approximately 95% after surgical resection, even among patients harboring aggressive disease characteristics [11].

Tumor burden reduction and potential prognostic benefit

In the context of metastatic disease, the resection of both the primary tumor and resectable metastases can offer significant benefits from both an oncological and a palliative perspective. Multiple studies indicate that debulking the primary mass and reducing the overall tumor burden can alleviate mass-effect symptoms, improve quality of life, and, in selected cases, prolong overall survival, even when SPN presents with synchronous hepatic metastases [8,10].

This cytoreductive approach is consistent with published reports where patients with metastatic SPN managed with aggressive surgical intervention achieved prolonged survival without significant clinical progression [12]. Although robust scientific evidence remains limited due to the rarity of this condition, the consensus across most clinical studies and expert opinions underscores that surgical resection remains the optimal therapeutic strategy whenever technically feasible [8].

Surgical decision-making considerations

The decision to perform an extensive resection, such as a distal pancreatectomy, portal vein thrombectomy, and a superior hemigastrectomy with esophagogastric reconstruction, requires a careful balance between surgical morbidity and potential prognostic benefits. In cases of SPN, even when presenting with advanced disease, the potential for long-term survival prolongation or clinical cure with an optimal quality of life justifies an aggressive surgical approach in highly selected patients [5].

Additionally, because SPN is generally characterized as a slow-growing neoplasm with a low proliferative index, removing the primary tumor and reducing the residual tumor volume supports the rationale that cytoreduction can mitigate the likelihood of aggressive clinical progression, even in the setting of concurrent metastatic disease.

The management of metastatic solid pseudopapillary neoplasms presents a significant clinical dilemma, particularly regarding the role of radical surgery in AJCC Stage IV disease. While aggressive multivisceral resection is often debated when distant metastases are present, the clinical indication in this case extended beyond theoretical cytoreduction. The patient's initial presentation was characterized by life-threatening gastrointestinal bleeding, manifested as severe melena and profound acute anemic syndrome, secondary to direct tumor invasion into the posterior gastric wall. Therefore, the surgical intervention served both an oncological and a vital palliative purpose. Addressing the local tumor burden through superior hemigastrectomy and distal esophagectomy was imperative to control the hemorrhagic focus and prevent imminent gastrointestinal perforation, transforming a high-risk cytoreductive procedure into a necessary, life-saving intervention [5,8].

Implications for clinical practice

This case reinforces that, in SPNs exhibiting aggressive clinical behavior, surgical intervention plays a pivotal curative role in localized disease and offers a potentially beneficial therapeutic impact in the metastatic setting. Even when a complete clinical cure is not achievable in advanced metastatic cases, current literature supports an aggressive surgical approach whenever feasible, as it can translate into prolonged overall survival and the alleviation of symptoms associated with tumor mass effect [5].

Clinical contribution and knowledge gaps

The clinical case presented herein provides additional evidence regarding the potential benefit of aggressive surgical resection of the primary tumor, even in patients with SPN harboring metastatic disease at the time of diagnosis. In the literature, SPNs are classically described as low-grade tumors with limited malignant potential, demonstrating high long-term survival rates following complete resection (5- to 10-year disease-free survival > 89-96%) and an indolent clinical course in most cases [5,13]. However, systematic reviews and multicenter studies detail that up to 10-15% of patients present with malignant features, including adjacent organ invasion or synchronous hepatic metastases at presentation [5,9,12].

Although radical surgery with negative margins is considered the gold standard and is associated with favorable oncological outcomes, the impact of resection in the presence of extensive metastatic disease and its potential to mitigate biological tumor progression has not been completely defined; this is primarily due to the rarity of this presentation and the lack of prospective comparative studies [5,9,14]. This case contributes to the growing body of evidence showing that an aggressive surgical strategy can favorably modify the clinical course, reduce overall tumor burden, and allow for prolonged survival, even in the presence of documented metastases [9,12].

Nevertheless, multiple gaps in the current knowledge of aggressive SPN persist. There is currently a lack of clear risk stratification criteria to determine which patients with metastatic pancreatic SPN will benefit most from extensive resections. Furthermore, prospective data evaluating the role of adjuvant therapies, highly specific prognostic biomarkers, and multimodal approaches in this disease remain scarce. Literature underscores the need for additional research to identify clinicopathological factors that can guide optimal therapeutic selection and to evaluate the role of complementary treatments to surgery in tumors presenting with local invasion or metastasis [14,15].

Following a successful R0 resection of the primary tumor and portal vein thrombectomy, the role of systemic adjuvant therapy was carefully evaluated. The case was reviewed comprehensively by a multidisciplinary tumor board. Due to the well-documented chemoresistance of solid pseudopapillary neoplasms and the current lack of standardized, high-level clinical evidence supporting systemic regimens for metastatic SPN, empirical chemotherapy or radiotherapy was withheld. Instead, a consensus was reached to pursue a strategy of strict clinical and radiological surveillance. This surveillance-guided approach aligns with observations by previous investigators who noted that aggressive surgical clearance of the primary tumor and local complications can yield prolonged survival and high functional status even in the presence of stable, residual metastatic disease, without exposing the patient to the added toxicity of empirical systemic treatments [5,9].

## Conclusions

Solid pseudopapillary neoplasms of the pancreas are generally characterized by an indolent clinical course; however, they can occasionally exhibit highly aggressive features, including extensive local invasion, vascular involvement, and synchronous distant metastases. This case demonstrates the technical feasibility of an aggressive, multivisceral surgical approach, driven by life-threatening local complications rather than pure cytoreductive intent, highlighting the potential to achieve clinical stability, functional preservation, and prolonged survival. Given the rarity of this presentation and the current gaps in risk stratification and adjuvant therapies, individualizing surgical decision-making remains paramount. Further prospective research is warranted to optimize multimodal management in this subset of patients.
